# Transcriptional Alterations Induced by Delta-9 Tetrahydrocannabinol in the Brain and Gonads of Adult Medaka

**DOI:** 10.3390/jox13020018

**Published:** 2023-05-30

**Authors:** Marlee Vassall, Sourav Chakraborty, Yashi Feng, Mehwish Faheem, Xuegeng Wang, Ramji Kumar Bhandari

**Affiliations:** 1 Department of Biology, University of North Carolina, Greensboro, NC 27412, USA; vassallmarlee@yahoo.com (M.V.); s_chakra@uncg.edu (S.C.);; 2 Institute of Modern Aquaculture Science and Engineering, College of Life Sciences, South China Normal University, Guangzhou 510631, China

**Keywords:** Δ^9^-THC, brain, medaka fish, reproductive effects, testis, ovary

## Abstract

With the legalization of marijuana smoking in several states of the United States and many other countries for medicinal and recreational use, the possibility of its release into the environment cannot be overruled. Currently, the environmental levels of marijuana metabolites are not monitored on a regular basis, and their stability in the environment is not well understood. Laboratory studies have linked delta 9-tetrahydrocannabinol (Δ^9^-THC) exposure with behavioral abnormalities in some fish species; however, their effects on endocrine organs are less understood. To understand the effects of THC on the brain and gonads, we exposed adult medaka (*Oryzias latipes*, Hd-rR strain, both male and female) to 50 ug/L THC for 21 days spanning their complete spermatogenic and oogenic cycles. We examined transcriptional responses of the brain and gonads (testis and ovary) to Δ^9^-THC, particularly molecular pathways associated with behavioral and reproductive functions. The Δ^9^-THC effects were more profound in males than females. The Δ^9^-THC-induced differential expression pattern of genes in the brain of the male fish suggested pathways to neurodegenerative diseases and pathways to reproductive impairment in the testis. The present results provide insights into endocrine disruption in aquatic organisms due to environmental cannabinoid compounds.

## 1. Introduction

Marijuana, a product of *Cannabis sativa*, has been used for centuries by various cultures throughout the world and consumed by an estimated 83 million individuals [1]. Due to its psychoactive properties, marijuana has been used extensively for the relief of nausea by pregnant women [2], and for treating drug-resistant epilepsy in children [3] as young as two months of age. The use of medical marijuana is legal in 33 states of the United States, while ten of them have approved its recreational use [4]. According to the National Survey on Drug Use and Health 2020, approximately 40% of Americans aged 12 and older have used marijuana for recreational purposes without considering its harmful effects [5]. This widespread use by young adults is raising significant health concerns. Marijuana, like other endocrine disrupting chemicals (EDCs), interacts with the endocrine system [6] at various levels to cause health hazards related to lifestyle.

The smoke of marijuana consists of around 400 chemicals, 150 of which are cannabinoid compounds. Cannabinoid receptors are present throughout the body, including the hypothalamus, pituitary [7], thyroid glands [8], uterus [9], ovary, testes, and sperm, as well as in the immune cells [10]. Therefore, it is crucial to understand the interaction of cannabinoid compounds present in marijuana with the body’s innate endocannabinoid system and study the impact on the physiological response. The primary bioactive constituent of marijuana is delta 9-tetrahydrocannabinol (Δ^9^-THC). It has an affinity to bind with specific G protein-coupled cannabinoid receptors; CB1 and CB2 [11]. It has been reported that THC exposure disrupts brain development by altering the transcriptional trajectory of prefrontal cortex (PFC) pyramidal neurons, changes hippocampal transcriptome, and alters different genes at transcriptional levels in the frontal and parietal cortex in adults [12,13] and promotes cognitive dysfunction [14]. Furthermore, THC impacts the levels of miRNA precursors and long non-coding RNAs in both total lymph node cells and CD4(+) T cells [15], promoting a compromised immunological state. Moreover, Δ^9^-THC has been associated with the dysregulation of microsomal oxidative metabolism [16]. According to studies, the CB1 receptor regulates food intake by modulating the release of corticotropin-releasing hormones and melanin-concentrating hormones [17,18]. Additionally, Δ^9^-THC is also involved in the dysregulation of the hypothalamic–pituitary–gonadal axis and the hypothalamic–pituitary–adrenal axis, promoting reproductive impairment such as oligomenorrhoea, lower libido, orgasm disorders, lack of ovulation, and a shortening of the luteal phase [19,20] in females and reduced fertilization rate in male [21]. THC exposure also promotes epigenetic modification in sperm [22], predicting transcriptional dysregulation in offspring. However, how THC exposure exerts transcriptional alterations in the reproductive tissues is currently unknown.

Δ^9^-THC has been found in surface water and influent wastewater. The reported concentrations in surface water range from 0.3–24 ng/L, while in influent wastewater, it has been reported in a range of 11.3–136 ng/L [23,24]. Since this substance is highly metabolized to THC-COOH, the parent THC compound is considerably low in the aquatic system [25]. The cannabis metabolite can exert an impact on the ecological species. Based on the activities of THC-COOH, which is a metabolite of THC, effects on aquatic fauna are expected. Studies on cannabinoid toxicity have been conducted on animals since 1960s [26], and studies were more concentrated on neuro and behavioral abnormalities [27,28] and adverse outcome pathways. Several studies have been conducted on zebrafish to study the developmental toxicity and teratogenic effects of THC in early neurogenesis [29], impaired memory function [30], psychosis [31], and embryo mortality [32]. The impact THC exposure on the brain and gonads of reproductively young adults is currently unknown. To understand this broader question, the present study, therefore, examined the transcriptional alterations in the gonads and brain of adult reproductively active male and female medaka after chronic exposure to Δ^9^-THC. Medaka, *Oryzias latipes*, was used as it processes epigenetic information in a similar fashion to mammals (humans and mice) [33], suggesting that the mechanistic molecular information obtained from the studies in medaka can be valuable for understanding epigenetic effects of Δ^9^-THC in aquatic species and other higher vertebrates, including humans.

## 2. Materials and Methods

### 2.1. Chemical Preparation

Δ^9^-tetrahydrocannabinol solution (1 mg/mL in methanol) was obtained from Sigma-Aldrich as a DEA Schedule II substance. Exposure solution of 50 μg/L was made by serially diluting commercially available THC pre-dissolved in 100% methanol. The final concentration of methanol in the dosing solution was 0.05% which was used as solvent control.

### 2.2. Animal Care and Exposure

The Hd-rR strain of medaka (*Oryzias latipes*) obtained from National BioResource Project, Japan, was used. The experiment was conducted using guidelines approved by the Institutional Animal Care and Use Committee at the University of North Carolina at Greensboro. The fish were maintained at the density of 3 males and 3 females per 4 L polysulfone tank. Fish were fed Otohime C1 medaka food (Reed Mariculture, Campbell, CA, USA). Since the environmental concentration of THC is currently unknown, a single concentration of 50 μg/L was selected to expose medaka for the present study, which is equivalent to the human plasma concentration of Δ^9^-THC (45 ng/mL) in chronic marijuana smokers [34]. Only two exposure groups were used (methanol solvent control and 50 μg/L Δ^9^-THC with three biological replicates per exposure group). The exposure was continued for 21 days, and the solutions were changed every 3 days.

### 2.3. Sample Collection, RNA Extraction, and RNA-Seq Library

After 21 days of exposure, fish were anesthetized with 250 mg/L MS-222, and brain and gonads were collected from male and female fish. Three biological replicates were used for the group, and each biological replicate contained 3 fish as technical replicates. In total, 36 brain samples, 18 testis samples, and 18 ovary samples were collected. RNA was extracted from each tissue sample using the Zymo Miniprep Plus Kit (Zymo Research, Irvine, CA, USA) with DNaseI digestion of RNA according to the manufacturer’s instructions. The quality control and RNA-seq library preparation were performed in-house using Qubit and Bioanalyzer.

### 2.4. RNA Sequencing (RNA-Seq) Data Analysis

Illumina HiSeq X Ten platform was used for RNA sequencing with the paired-end sequencing strategy of 150 bp. The sequencing output was 10–15 million reads per minute per sample, with an alignment rate of 89%. The reads were analyzed using our in-house RNA seq data analysis protocol, and differentially expressed genes were determined using Cuffdiff. Fragments per kilobase of transcript per million mapped fragments (FPKM) were normalized for the calculation of the expression value of genes. Genes were considered differentially significant with the FDR-adjusted *p*-value < 0.05 (q-value). The differentially expressed genes were presented as the control and Δ^9^-THC-exposed group. All the sequencing data have been deposited to NCBI Gene Expression Omnibus (GEO) with the accession number GSE129727.

### 2.5. Gene Ontology Analyses

GO terms associated with Δ^9^-THC exposure were searched using the g:profiler tool [35]. Ensembl gene data were used to map medaka genes of interest to their human orthologs in order to take full advantage of the many functional and non-inferred electronic annotations based on the human genome [36]. Using the online g-profiler ortholog search tool, we converted the Ensembl medaka genome ID into a human ID. Programs, mainly g:profiler and Enrichr were used to analyze the transcriptome data, ontology, human phenotype, Reactome, KEGG, and transcription factors based on gene lists. To ensure that only the most relevant terms were selected, we applied Bonferroni FDR correction. A GO term with a corrected *p* value of 0.05 or less was considered significantly enriched among differentially expressed genes (DEGs).

### 2.6. qRT-PCR

RNA isolated in step 2.3 was used to make cDNA using M-MLV reverse transcriptase enzyme system (ThermoFisher Scientific, Waltham, MA, USA) according to the manufacturer’s instructions as previously described [37]. A real-time qPCR analysis was conducted using gene-specific primers. Primers were designed by using Primer3web from exon-exon junctions to avoid genomic DNA amplification. After testing the expression pattern of several housekeeping genes, 18s rRNA was selected as a stable housekeeping gene. The fold change was analyzed with the 2^−∆∆Ct^ method.

## 3. Results

To detect gene expression differences between exposed and control fish after a 21-day exposure to THC (50 μg/L), we performed RNA sequencing of brain and gonad samples of both adult THC-exposed and control fish. The raw data were analyzed based on the previous tool [38]. Only statistically significant genes (q < 0.05) were considered for further analysis. The human ortholog of medaka genes was determined (Supplementary Figure S1) and used for enrichment analysis. Differential expression of genes was found to be enriched in the male brain and testis. In females, only the brain showed differential gene expression patterns but not the ovaries.

### 3.1. Transcriptomic Changes in the Male Brain

**Figure 1:**
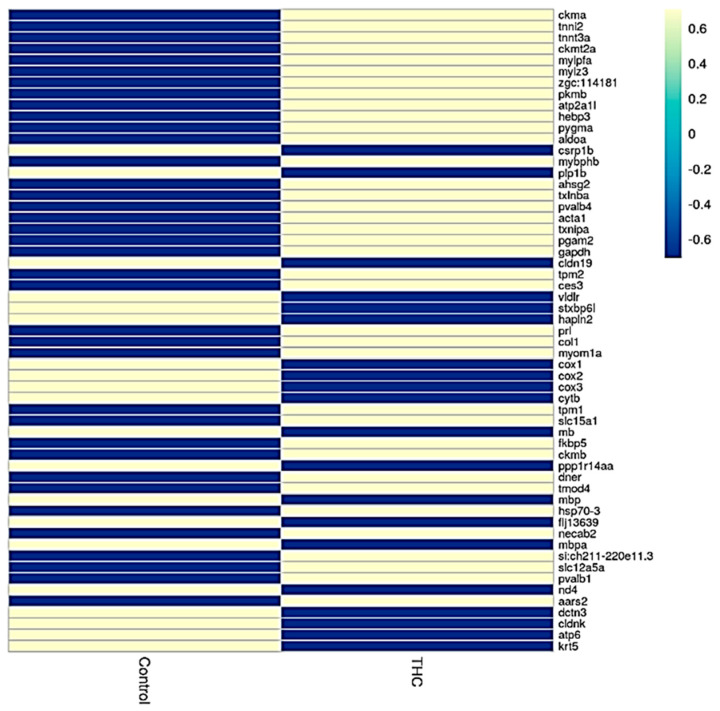
Heatmap analysis showing expression of genes in control and Δ^9^-THC-exposed male brain.

**Figure 2:**
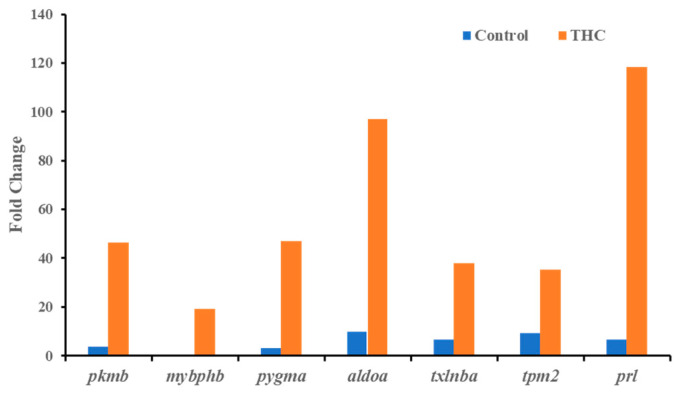
Highly upregulated genes (Δ^9^-THC-specific biomarkers) in the male brain.

**Figure 3:**
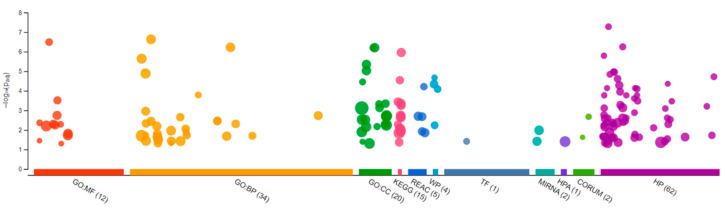
Gene Ontology analysis showing differentially expressed genes in the brain of the Δ^9^-THC-exposed males. A total of 12 molecular functions, 34 biological processes, and 20 cellular components were found to be differentially affected.

**Figure 4:**
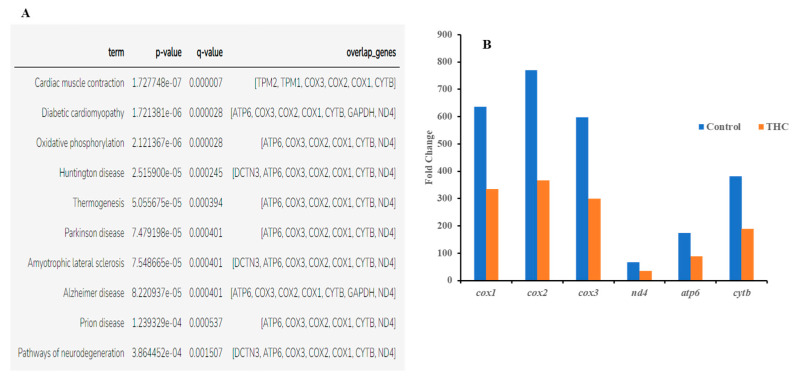
(**A**) Enriched disease-associated DEGs from male brain DEG list (q < 0.05 and below). (**B**) Common DEGs found in all disease phenotypes.

Sequencing of the male brain RNA revealed 57 significant DEGs (Figure 1). Additionally, highly upregulated DEGs were identified as biomarkers (Figure 2) from total DEGs. A total of 50 human orthologs of medaka were examined for their biological relationship using enrichment analysis based on DEGs. In the male brain, 12 molecular functions (MF), 34 biological processes (BP), and 20 cellular functional (CF) enrichments were identified (Figure 3). The top 12 enrichments from each category were chosen to illustrate the biological function of the DEGs. Structural constituent of muscle (GO:0008307 q = 3.24E−07), redox-driven active transport muscle (GO:0015453 q = 3.07E−04), cytochrome-c oxidase activity (GO:0004129 q = 4.34E−03) are mostly enriched in molecular function (Supplementary Figure S2A). ATP metabolic process (GO:0046034 q = 5.97E−07), muscle contraction (GO:0006936 q = 2.33E−07), oxidative phosphorylation (GO:0006119 q = 1.102E−03), and actin-myosin filament sliding (GO:0033275 q = 1.63E−04) are the highest enrichments in biological processes (Supplementary Figure S2B). Respiratory chain complex III and IV, cytochrome complex, and respirasome are the highest enrichments in cellular components (Supplementary Figure S2C). In pathways enrichment analysis, unclassified pathways are altered greater than the other enriched pathways. Most genes associated with mitochondrial dysregulation, such as cox1, cox2, cox3, nd4, atp6, and cytb, are involved in various common diseases (Figure 4).

**Figure 5:**
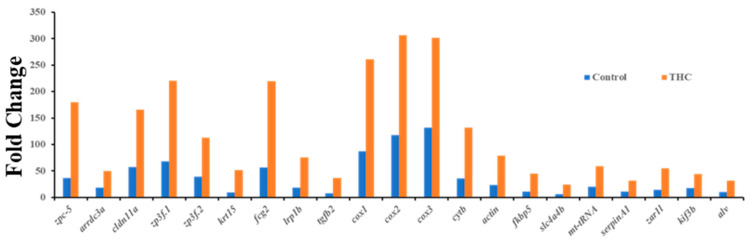
Differentially expressed genes (upregulated) in the testis (q < 0.05 or below).

**Figure 6:**
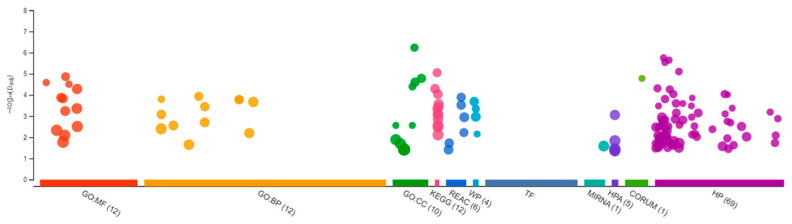
Gene Ontology showing differentially expressed genes in the testis of the Δ^9^-THC-exposed males. A total of 12 genes were enriched in molecular function, 12 in biological processes, and 10 in cellular components, with additional pathways enrichment found in the brain of the Δ^9^-THC-exposed female medaka.

In the testis, the expression of 22 genes was significantly altered. Many DEGs were upregulated (Figure 5). Human orthologues of genes showed the genes that regulate hormones, embryonic development, and energy uptake. Gene Ontology annotation was performed on the 22 genes to obtain their biological roles, and MF (12), BP (12), and CF (10) were found (Figure 6). Molecular function of the top 12 enriched genes are presented, namely, oxidoreduction-driven active transmembrane transporter (GO:0004129 q = 2.60E−05), respiratory electron transport chain (GO:0015453 q = 1.36E−05), cytochrome-c oxidase activity (GO:0004129 q = 2.60E−05), electron transfer activity (GO:0015453 q = 1.36E−05), ATP synthesis coupled electron transport, female gamete generation, and fertilization are the highest enrichments (Supplementary Figure S3A). In molecular functions, oxidoreduction-driven active transmembrane transporter heme-copper terminal oxidase activity, cytochrome-c oxidase activity, oxidoreductase activity, and electron transfer activity were the highly enriched functions to be affected by THC exposure (Supplementary Figure S3B).

### 3.2. Transcriptomic Changes in Females

**Figure 7:**
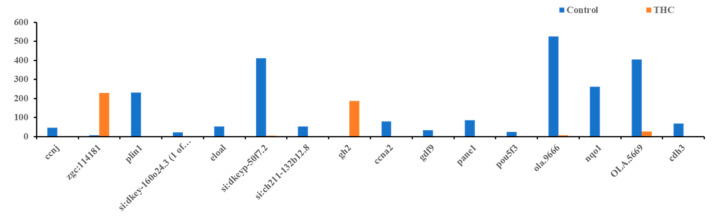
Differentially expressed genes in the female brain. Bars above zero are upregulated (q < 0.05 and below).

**Figure 8:**
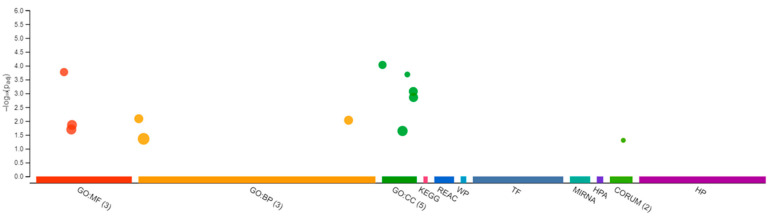
Differentially expressed genes in Gene Ontology from Δ^9^-THC-exposed adult female medaka brain. In total, 3 in molecular function, 3 biological processes, and 5 cellular components with additional pathways enrichment were found in THC-exposed brain from medaka.

The RNA sequencing of the female brain revealed 22 significantly differentially expressed genes, of which 06 were unidentified genes and 16 were annotated. Most DEGs were downregulated except gh2 and zgc:114181 (Figure 7). Among the 16 genes, eloal and pou5f3 are specific to fish, and the rest were conserved with humans. The 22 human orthologs of medaka genes from female brains were used to perform Gene Ontology annotation. The biological roles and significant enrichment were determined (Figure 8). Regulation of cyclin-dependent protein serine/threonine kinase activity (GO:0016538, q = 2.58E−04), regulation of cyclin-dependent protein kinase activity (GO:0019887, q = 2.09E−02) were most significant under molecular function (Supplementary Figure S4A). Regulation of protein phosphorylation (GO:1904029, q = 1.39E−02), mitotic cell cycle phase transition, and cell cycle phase transition (GO:0044772, q = 4.18E−02) were the highest enrichment in biological processes (Supplementary Figure S4B). Protein kinase complex, serine/threonine protein kinase complex (GO:1902554, q = 1.09E−03), cyclin A2-CDK2 complex (GO:0097124, q = 2.36E−04), and cyclin-dependent protein kinase holoenzyme complex (GO:0000307, q = 1.20E−04) were the highest enrichments of cellular components (Supplementary Figure S4C). It was also identified that DEGs in male and female brains were mostly unique (Supplementary Figure S5). Interestingly, no DEGs were found in the RNA collected from ovaries.

### 3.3. qRT-PCR Results

**Figure 9:**
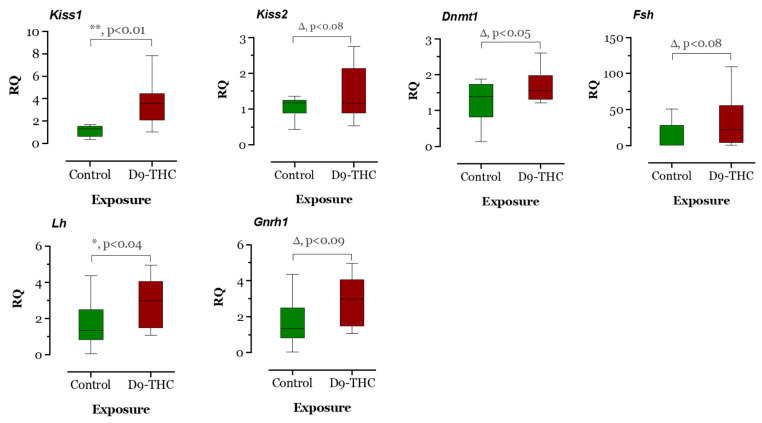
Gene expression analysis of selected genes in the female brain. Floating bars show values with standard deviation from the mean. Asterisks indicate the statistical significance and *p* values *p* ≤ 0.05.

**Figure 10:**
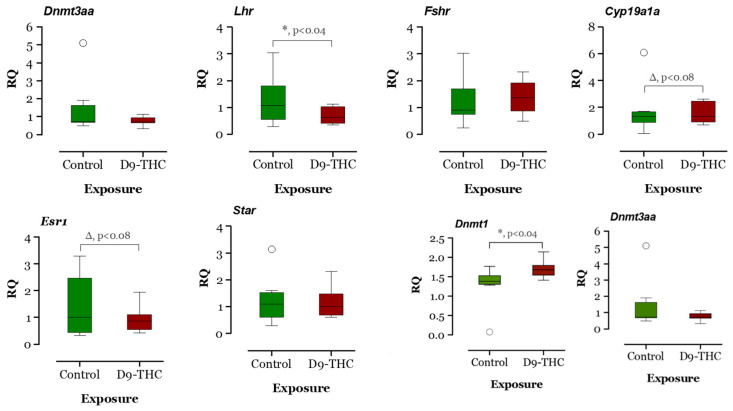
Gene expression analysis of selected genes in the female ovary. Floating bars show values with standard deviation from the mean. Asterisks indicate the statistical significance and *p* values *p* ≤ 0.05.

**Figure 11:**
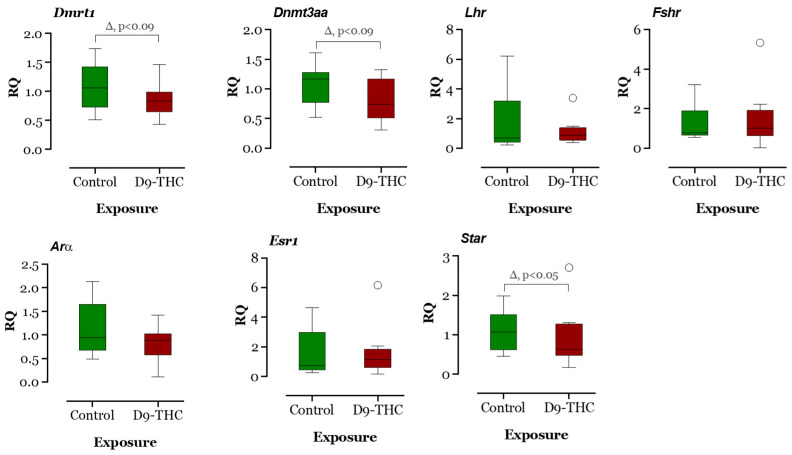
Gene expression analysis of selected genes in the male testis. Floating bars show values with standard deviation from the mean. Asterisks indicate the statistical significance and *p* values *p* ≤ 0.05.

The expression of DNA methyltransferase genes in the Δ^9^-THC-exposed female brain, ovary, and testis were examined using qRT-PCR. In the Δ^9^-THC-exposed female brain and ovary, the *dnmt1* gene was expressed significantly higher than the control group (*p* < 0.05, Figure 9 and Figure 10), while *dnmt3aa* was significantly decreased in ovaries (Figure 10). In the testis, only *dnmt3aa*, *dmrt1*, and *star* genes showed a significant decrease in expression compared to the control (Figure 11). In the ovary, *esr1* and *lhr* were significantly decreased, while *cyp19a1a* was significantly increased in the THC group in comparison to the control (Figure 10). In the brain, *kiss1*, *kiss2*, *dnmt1*, *lh*, and *gnrh1* genes were significantly upregulated compared to the control brains (Figure 9). The *lh* mRNAs were measured as the pituitary was mixed with brain samples to avoid loss of RNA from pituitary gland because of their size.

## 4. Discussion

Multiple pathways of toxicity can be enhanced by environmental chemicals leading to adverse health outcomes either immediately or later in life [39]. Mammalian endogenous cannabinoid receptors are known to be affected by the frequent use of cannabinoids [40,41], while the effects of Δ^9^-THC on aquatic organisms remain ambiguous. Thus, the goal of the present study was to investigate whether Δ^9^-THC can affect the brain and reproductive tissues of adult fish taking a transcriptomics approach. RNAseq technology is a powerful tool to determine the expression levels of thousands of genes simultaneously in various tissues. Transcriptomic studies of the brain and gonads of both sexes of medaka fish reflected the neurotoxic and reproductive effects of Δ^9^-THC exposure, suggesting the effects of THC on reproductive and behavioral manifestations in aquatic organisms.

In the present study, RNA sequencing data showed that the expression of *cox1*, *cox2*, *cox3*, *nd4*, *atp6*, and *cytb* genes are significantly downregulated in the male brain, indicating adult exposure to THC may induce neurodegenerative disorder in males. Additionally, *tmod4*, *tpm1*, *tpm2*, *col1*, *acta1*, *hsp70*, *aldoa*, and *fkbp5* are significantly upregulated in the male brain after Δ^9^-THC exposure. Most information related to Δ^9^-THC effects on the brain has been centered on mammalian studies and cell cultures. No information is available regarding chronic exposure effects on adult male brains and other reproductive tissues in aquatic species. The male mice chronically exposed to Δ^9^-THC developed long-term cognitive and behavioral disorders [42]. In various animal models, it has been found that Δ^9^-THC is involved in regulating oxidative stress/mitochondrial functioning and cytoarchitecture [43] in the brain. As the brain is a highly glucose-consuming vital organ, the rate of oxidative phosphorylation and the rate of ATP production is higher compared to other organs. Downregulation of genes encoding various proteins for glucose metabolism and oxidative phosphorylation is correlated with various diseases such as Alzheimer [44]. In the literature, it is suggested that decreased mitochondrial cytochrome C oxidase (*cox*) mRNA levels may indicate the intensity of synaptic activity, Parkinson’s [45], and Huntington’s diseases [46]. In contrast, the downregulation of NADH dehydrogenase subunit genes such as *nd4* can be associated with neurodegenerative disease [47]. A previous study with zebrafish reported downregulation of the *cox* gene to be correlated with epilepsy [48]. Upregulation of *aldoa* leads to impaired glucose metabolism in the brain, which may result in Alzheimer’s disease [49]. Studies with mice and zebrafish reported that Δ^9^-THC exposure could cause oxidative stress with an increased expression of *fkbp5* and *hsp70* genes in the brain [50]. Taken together, the present transcriptomic results suggested the overall neurotoxicity of Δ^9^-THC exposure in fish.

Based on the Gene Ontology annotations, genes that contribute to the enrichment of biological processes are mainly related to cell respiratory pathways and muscle contraction pathways. Genes encoding enzymes that participate in glycolysis and the genes encoding enzymes that contribute to the electron transport chain were all upregulated by THC. Research suggests that Δ^9^-THC exposure is associated with hypothermia and mitochondrial function disruption, resulting in energy depletion [51,52]. It is, therefore, suggested that the mitochondrial respiratory chain could be impaired, leading to mitochondrial dysfunction as well as increased oxidative stress [53] in Δ^9^-THC-exposed male medaka brain.

In the present study, highly significant DEGs were screened out from the female brain of the THC exposure group compared to the control. Genes such as *ccnj* (regulating cell cycles), *plin1*, (lipid droplet-associated protein), *eloa1* (transcriptional factor), *ccna2* (cell cycle regulator), *gdf9* (development of primary follicles in the ovary), and *nqo1* (regulates cell cycles) were significantly downregulated in Δ^9^-THC-exposed female brain transcriptomes. However, the *dnmt1* and *kiss* genes were significantly upregulated in the brain of THC-exposed females. The Δ^9^-THC exposure can alter gene expression patterns and promote dysregulation of neuronal, astrocytic, and microglial signaling pathways in mice [50]. A study showed reduced dorsal hippocampal neurogenesis linked to impaired spatial memory in male rats promoted by chronic exposure to the synthetic cannabinoid [54]. Neurogenesis is closely controlled by various factors, including transcription factors, cytokines, and trophic factors [14,55], especially the cyclin-CDK complex [56]. The application of the CDK inhibitor in the cell cycle arrest phase of neurons reflects the intervention of cell cycle genes in neuronal differentiation and proliferation [56]. The *ccna2* gene contributes to two essential control points during the cell cycle by binding to cyclin-dependent kinases (CDKs) [57]. It binds to CDK2 during the S phase and CDK1 during the G2 phase. The ccna2-cdk2 complex is suggested to act during the initiation and progression of DNA synthesis, preventing the re-replication of DNA during the cell cycle. The *eloa1* gene encodes protein elongin A, which enhances the RNA Pol II elongation rate [58], demonstrating that downregulation of *eloa1* may also restrict neurogenesis. Additionally, meta-analysis of the prognostic significance of plin1 has shown that the downregulation of the plin1 gene is directly linked with tumorigenesis and metastasis [59]. In rat brains, cannabinoids increase the activity of *dnm1* [60], resulting in the alteration of methylome patterns. Thus, upregulation of *dnmt1* in the Δ^9^-THC-exposed medaka brain may alter the brain methylome profile, which could result in the downregulation of genes. To understand how those genes are differentially expressed in female brains, a comprehensive genome-wide epigenetic and simultaneous transcriptomic mapping is needed, which future studies will address. The *ccnj* and *ccnp* genes are not characterized in medaka fish, while *ccna2* is predicted to be expressed in dividing somatic cells and controlling the cell cycle [61]. All the cyclin genes and *plin1* genes are downregulated in the Δ^9^-THC-exposed female group, suggesting that the cell cycle checkpoints of somatic cell cycles are possibly disrupted, and abnormal cell growth might be noticeably induced, leading to the development of cancer cells. In addition, the sequencing analysis uncovered GO terms that strengthen the connection between Δ^9^-THC exposure and the development of adverse outcomes in female brains. Analysis of the RNA-Seq data determined that exposure to Δ^9^-THC enriched the same set of genes associated with changes in the regulation of cyclin-dependent protein serine/threonine kinase regulation and is supported by previous studies associating dysregulation in the cell cycle that leads to metastasis [62,63].

The present results showed downregulation of *dnmt3aa* and upregulation of *zpc5*, *arrdc3a*, *cldn11a*, *krt15*, *fcg2*, *tgfb2*, *cox*, and *cytb* expression. In medaka, the upregulation of these genes could be correlated with the downregulation of *dnmt3aa*, indicating epigenetic control of Δ^9^-THC exposure on testicular gene expression. In mammals, altered endocannabinoid signaling has been reported to cause male reproduction disorders by inhibiting spermatogenesis, reducing gonadotropin levels, and affecting the appearance of sperm [64] due to the direct Δ^9^-THC exposure effect. As suggested by the literature, downregulation of *cox* and *cytb* genes in testes, which control energy metabolism, can be associated with testicular atrophy [65], while upregulation of *cox* genes can produce proinflammatory cytokines and reactive oxygen species which are responsible for the development of several types of cancers [66], illustrating that adult Δ^9^-THC exposure might have developed oxidative stress in medaka testis, which may lead to several reproductive diseases. The zona pellucida (ZP) is found in developing oocytes. A study found that *zpc5* upregulation in the testis indicates the formation of the testis–ova complex in medaka [67], which suggests that *zpc5* upregulation in the testis of adult Δ^9^-THC-exposed males could impair male reproduction. In THC-exposed males, downregulation of *dmrt1* indicates a possible disruption of spermatogenesis in the testis, and downregulation of *star* further supports this notion that a disruption in testosterone synthesis could be occurring in these Δ^9^-THC-exposed medaka [68]. The result of RNAseq for testis shows that there are 22 differentially expressed genes, of which only 14 genes are annotated. Among the 14 genes, there are 4 genes related to metabolism: *cox1*, *cox2*, *cox3*, and *cytb*. These genes are responsible for energy production through aerobic metabolism [69], although the *cox3* gene is not functional in humans. Like *cox* genes, the *cytb* gene is a cytochrome b gene that functions as part of the electron transport chain [70]. All these metabolism-related genes are upregulated to at least 1.2-fold in THC-exposed testes. The Gene Ontology annotations suggest that the pathways related to the respiratory electron transport chain are altered due to alterations in the expression of the aforementioned genes. This is consistent with Gene Ontology analysis of the male brain, which suggest that Δ^9^-THC targeted mainly metabolism and respiratory aspects in male fish. The alterations of cytochrome c subunit genes and cytochrome b genes are aligned with the finding that THC impairs mitochondrial function and results in hypothermia in organisms [52,53,71]. Based on these findings, it is reasonable to suggest that THC targets respiratory chains and metabolism in the male testis.

## 5. Conclusions

In this study, we investigated the effects of human-relevant Δ^9^-THC exposure in adult male and female medaka. Transcriptome analyses of the male brain exposed to Δ^9^-THC revealed that the THC exposure altered the expression of genes related to mitochondrial dysfunction, oxidative phosphorylation, and enriched gene sets identified in Parkinson’s and Alzheimer’s disease. Additionally, abnormal expression of cell-cycle-controlling genes identified in the female brain are potentially associated with the metastasis pathway. The male reproductive system, mainly at the gonadal level, can be affected by chronic Δ^9^-THC exposure, which is also supported by the enriched DEGs in several biological functions. Interestingly, no significant DEGs were found in the ovaries in females, suggesting a sexually dimorphic action of Δ^9^-THC in the gonads. Overall, this study will open new arenas of research to understand the role of THC in brain metabolism and reproduction in higher mammals, including humans, using fish as a plausible animal model.

## Data Availability

Data are contained within the article or supplementary material. The data presented in this study are available on request from the corresponding author.

## Supplementary Materials

The following supporting information can be downloaded at: https://www.mdpi.com/article/10.3390/jox13020018/s1, Figure S1: The human ortholog of medaka genes in the male brain, testis, and female brain. Y axis indicates the number of differentially expressed genes enriched.; Figure S2: The top 12 enrichments of the biological processes from the Gene Ontology enrichment annotation of male brain from THC-exposed medaka. (2A) Enrichment of biological processes. (2B) Enrichment of molecular function. (2C) Enrichment of cellular components; Figure S3: Top 12 enrichments of the biological processes from the Gene Ontology enrichment annotation of testis from Δ9-THC -exposed medaka. (3A) Enrichment of biological processes. (3B) Enrichment of molecular function. (3C) Enrichment of cellular components; Figure S4: Gene Ontology enrichment annotation of the female brain. (4A) Enrichment of biological processes. (4B) Enrichment of molecular function. (4C) Enrichment of cellular components; Figure S5: Venn diagram of DEG in males and females showed a unique set of genes differentially expressed in THC-exposed medaka.

## References

[1] Maccarrone M., Rapino C., Francavilla F., Barbonetti A. (2021). Cannabinoid signaling and effects of cannabis on the male reproductive system. Nat. Rev. Urol..

[2] Parker L.A., Rock E.M., Limebeer C.L.J. (2011). Regulation of nausea and vomiting by cannabinoids. Br. J. Pharmacol..

[3] Wu Y.W., Sullivan J., McDaniel S.S., Meisler M.H., Walsh E.M., Li S.X., Kuzniewicz M.W. (2015). Incidence of Dravet Syndrome in a US Population. Pediatrics.

[4] Yu B., Chen X., Chen X., Yan H. (2020). Marijuana legalization and historical trends in marijuana use among US residents aged 12–25: Results from the 1979–2016 National Survey on drug use and health. BMC Public Health.

[5] Leghissa A., Hildenbrand Z.L., Schug K.A. (2019). The imperatives and challenges of analyzing Cannabis edibles. Curr. Opin. Food Sci..

[6] Komorowski J., Stepień H. (2007). The role of the endocannabinoid system in the regulation of endocrine function and in the control of energy balance in humans. Postep. Hig. I Med. Dosw..

[7] Berdyshev E.V. (2000). Cannabinoid receptors and the regulation of immune response. Chem. Phys. Lipids.

[8] Rodríguez de Fonseca F., Navarro M., Gómez R., Escuredo L., Nava F., Fu J., Murillo-Rodríguez E., Giuffrida A., LoVerme J., Gaetani S. (2001). An anorexic lipid mediator regulated by feeding. Nature.

[9] Burdyga G., Lal S., Varro A., Dimaline R., Thompson D.G., Dockray G.J. (2004). Expression of cannabinoid CB1 receptors by vagal afferent neurons is inhibited by cholecystokinin. J. Neurosci. Off. J. Soc. Neurosci..

[10] Porcella A., Marchese G., Casu M.A., Rocchitta A., Lai M.L., Gessa G.L., Pani L. (2002). Evidence for functional CB1 cannabinoid receptor expressed in the rat thyroid. Eur. J. Endocrinol..

[11] Dennedy M.C., Friel A.M., Houlihan D.D., Broderick V.M., Smith T., Morrison J.J. (2004). Cannabinoids and the human uterus during pregnancy. Am. J. Obstet. Gynecol..

[12] Jean-Gilles L., Braitch M., Latif M.L., Aram J., Fahey A.J., Edwards L.J., Robins R.A., Tanasescu R., Tighe P.J., Gran B. (2015). Effects of pro-inflammatory cytokines on cannabinoid CB 1 and CB 2 receptors in immune cells. Acta Physiol..

[13] Schatz A.R., Lee M., Condie R.B., Pulaski J.T., Kaminski N.E. (1997). Cannabinoid receptors CB1 and CB2: A characterization of expression and adenylate cyclase modulation within the immune system. Toxicol. Appl. Pharmacol..

[14] Leishman E., Murphy M., Mackie K., Bradshaw H.B. (2018). Δ(9)-Tetrahydrocannabinol changes the brain lipidome and transcriptome differentially in the adolescent and the adult. Biochim. Biophys. Acta Mol. Cell Biol. Lipids.

[15] Miller M.L., Chadwick B., Dickstein D.L., Purushothaman I., Egervari G., Rahman T., Tessereau C., Hof P.R., Roussos P., Shen L. (2019). Adolescent exposure to Δ9-tetrahydrocannabinol alters the transcriptional trajectory and dendritic architecture of prefrontal pyramidal neurons. Mol. Psychiatry.

[16] Renard J., Krebs M.O., Le Pen G., Jay T.M. (2014). Long-term consequences of adolescent cannabinoid exposure in adult psychopathology. Front. Neurosci..

[17] Yang X., Bam M., Nagarkatti P.S., Nagarkatti M. (2016). RNA-seq analysis of δ9-tetrahydrocannabinol-treated T cells reveals altered gene expression profiles that regulate immune response and cell proliferation. J. Biol. Chem..

[18] Narimatsu S., Watanabe K., Matsunaga T., Yamamoto I., Imaoka S., Funae Y., Yoshimura H. (1992). Cytochrome P-450 isozymes involved in the oxidative metabolism of delta 9-tetrahydrocannabinol by liver microsomes of adult female rats. Drug Metab. Dispos..

[19] Cota D., Marsicano G., Lutz B., Vicennati V., Stalla G.K., Pasquali R., Pagotto U. (2003). Endogenous cannabinoid system as a modulator of food intake. Int. J. Obes..

[20] Rutkowska M., Jamontt J. (2005). Involvement of the Cannabinoid System in the Regulation of Food Intake. Adv. Clin. Exp. Med..

[21] Mendelson J.H., Mello N.K., Ellingboe J., Skupny A.S., Lex B.W., Griffin M. (1986). Marihuana smoking suppresses luteinizing hormone in women. J. Pharmacol. Exp. Ther..

[22] Schuel H., Goldstein E., Mechoulam R., Zimmerman A.M., Zimmerman S. (1994). Anandamide (arachidonylethanolamide), a brain cannabinoid receptor agonist, reduces sperm fertilizing capacity in sea urchins by inhibiting the acrosome reaction. Proc. Natl. Acad. Sci. USA.

[23] Carvalho R.K., Souza M.R., Santos M.L., Guimarães F.S., Pobbe R.L.H., Andersen M.L., Mazaro-Costa R. (2018). Chronic cannabidiol exposure promotes functional impairment in sexual behavior and fertility of male mice. Reprod. Toxicol..

[24] Murphy S.K., Itchon-Ramos N., Visco Z., Huang Z., Grenier C., Schrott R., Acharya K., Boudreau M.H., Price T.M., Raburn D.J. (2018). exposure and altered DNA methylation in rat and human sperm. Epigenetics.

[25] Boleda M.R., Galceran M.T., Ventura F. (2009). Monitoring of opiates, cannabinoids and their metabolites in wastewater, surface water and finished water in Catalonia, Spain. Water Res..

[26] How Z.T., Gamal El-Din M. (2021). A critical review on the detection, occurrence, fate, toxicity, and removal of cannabinoids in the water system and the environment. Environ. Pollut..

[27] Postigo C., de Alda M.J.L., Barceló D. (2010). Drugs of abuse and their metabolites in the Ebro River basin: Occurrence in sewage and surface water, sewage treatment plants removal efficiency, and collective drug usage estimation. Environ. Int..

[28] Peng H., Li H., Wei Y., Zhang R., Chang X., Meng L., Wang K., He Q., Duan T. (2023). Effects of prenatal exposure to THC on hippocampal neural development in offspring. Toxicol. Lett..

[29] Shollenbarger S.G., Price J., Wieser J., Lisdahl K. (2015). Impact of cannabis use on prefrontal and parietal cortex gyrification and surface area in adolescents and emerging adults. Dev. Cogn. Neurosci..

[30] Reece A.S. (2020). Canadian cannabis consumption and patterns of congenital anomalies: An ecological geospatial analysis. J. Addict. Med..

[31] Persaud T., Ellington A. (1968). Teratogenic activity of cannabis resin. Lancet.

[32] Becker B., Wagner D., Gouzoulis-Mayfrank E., Spuentrup E., Daumann J. (2010). Altered parahippocampal functioning in cannabis users is related to the frequency of use. Psychopharmacology.

[33] Stewart A.M., Kalueff A.V. (2014). The behavioral effects of acute Δ9-tetrahydrocannabinol and heroin (diacetylmorphine) exposure in adult zebrafish. Brain Res..

[34] Carty D.R., Thornton C., Gledhill J.H., Willett K.L. (2018). Developmental Effects of Cannabidiol and Δ9-Tetrahydrocannabinol in Zebrafish. Toxicol. Sci..

[35] Ruhl T., Prinz N., Oellers N., Seidel N.I., Jonas A., Albayram Ö., Bilkei-Gorzo A., von der Emde G. (2014). Acute administration of THC impairs spatial but not associative memory function in zebrafish. Psychopharmacology.

[36] Dahlén A., Zarei M., Melgoza A., Wagle M., Guo S. (2021). THC-induced behavioral stereotypy in zebrafish as a model of psychosis-like behavior. Sci. Rep..

[37] Thomas R. (1975). The toxicologic and teratologic effects of Δ9-tetrahydrocannabinol in the Zebrafish embryo. Toxicol. Appl. Pharmacol..

[38] Wang X., Bhandari R.K. (2020). The dynamics of DNA methylation during epigenetic reprogramming of primordial germ cells in medaka (*Oryzias latipes*). Epigenetics.

[39] Couper F.J., Logan B.K. (2014). Drugs and Human Performance Fact Sheets (DOT HS 809 725). Natl. Highw. Traffic Saf. Adm..

[40] Raudvere U., Kolberg L., Kuzmin I., Arak T., Adler P., Peterson H., Vilo J. (2019). g:Profiler: A web server for functional enrichment analysis and conversions of gene lists (2019 update). Nucleic Acids Res..

[41] Herrero J., Muffato M., Beal K., Fitzgerald S., Gordon L., Pignatelli M., Vilella A.J., Searle S.M.J., Amode R., Brent S. (2016). Ensembl comparative genomics resources. Database.

[42] Wang X., Hill D., Tillitt D.E., Bhandari R.K. (2019). Bisphenol A and 17α-ethinylestradiol-induced transgenerational differences in expression of osmoregulatory genes in the gill of medaka (*Oryzias latipes*). Aquat. Toxicol..

[43] Bhandari R.K., vom Saal F.S., Tillitt D.E. (2015). Transgenerational effects from early developmental exposures to bisphenol A or 17α-ethinylestradiol in medaka. Oryzias latipes. Sci. Rep..

[44] Angrish M.M., Allard P., McCullough S.D., Druwe I.L., Helbling Chadwick L., Hines E., Chorley B.N. (2018). Epigenetic applications in adverse outcome pathways and environmental risk evaluation. Environ. Health Perspect..

[45] Di Franco N., Drutel G., Roullot-Lacarrière V., Julio-Kalajzic F., Lalanne V., Grel A., Leste-Lasserre T., Matias I., Cannich A., Gonzales D. (2022). Differential expression of the neuronal CB1 cannabinoid receptor in the hippocampus of male Ts65Dn Down syndrome mouse model. Mol. Cell. Neurosci..

[46] Reitsma S.E., Lakshmanan H.H.S., Johnson J., Pang J., Parra-Izquierdo I., Melrose A.R., Choi J., Anderson D.E., Hinds M.T., Stevens J.F. (2022). Chronic edible dosing of Δ9-tetrahydrocannabinol (THC) in non-human primates reduces systemic platelet activity and function. Am. J. Physiol. Cell Physiol..

[47] Murphy M., Mills S., Winstone J., Leishman E., Wager-Miller J., Bradshaw H., Mackie K. (2017). Chronic adolescent Δ9-tetrahydrocannabinol treatment of male mice leads to long-term cognitive and behavioral dysfunction, which are prevented by concurrent cannabidiol treatment. Cannabis Cannabinoid Res..

[48] Quinn H.R., Matsumoto I., Callaghan P.D., Long L.E., Arnold J.C., Gunasekaran N., Thompson M.R., Dawson B., Mallet P.E., Kashem M.A. (2008). Adolescent Rats Find Repeated Δ9-THC Less Aversive Than Adult Rats but Display Greater Residual Cognitive Deficits and Changes in Hippocampal Protein Expression Following Exposure. Neuropsychopharmacology.

[49] Chandrasekaran K., Hatanpää K., Brady D.R., Rapoport S.I. (1996). Evidence for Physiological Down-regulation of Brain Oxidative Phosphorylation in Alzheimer’s Disease. Exp. Neurol..

[50] Teismann P. (2012). COX-2 in the neurodegenerative process of Parkinson’s disease. BioFactors.

[51] Kumar P., Kalonia H., Kumar A. (2011). Role of LOX/COX pathways in 3-nitropropionic acid-induced Huntington’s Disease-like symptoms in rats: Protective effect of licofelone. Br. J. Pharmacol..

[52] Fukuyama R., Hatanpää K., Rapoport S.I., Chandrasekaran K. (1996). Gene expression of ND4, a subunit of complex I of oxidative phosphorylation in mitochondria, is decreased in temporal cortex of brains of Alzheimer’s disease patients. Brain Res..

[53] Barbalho P.G., Lopes-Cendes I., Maurer-Morelli C.V. (2016). Indomethacin treatment prior to pentylenetetrazole-induced seizures downregulates the expression of il1b and cox2 and decreases seizure-like behavior in zebrafish larvae. BMC Neurosci..

[54] Muller T., Jung K., Ullrich A., Schrotter A., Meyer H., Stephan C., Egensperger R., Marcus K. (2008). Disease state, age, sex, and post-mortem time-dependent expression of proteins in AD vs. control frontal cortex brain samples. Curr. Alzheimer Res..

[55] Chiu P., Karler R., Craven C., Olsen D., Turkanis S. (1975). The influence of delta9-tetrahydrocannabinol, cannabinol and cannabidiol on tissue oxygen consumption. Res. Commun. Chem. Pathol. Pharmacol..

[56] Sarafian T.A., Kouyoumjian S., Khoshaghideh F., Tashkin D.P., Roth M.D. (2003). Delta 9-tetrahydrocannabinol disrupts mitochondrial function and cell energetics. Am. J. Physiol. Lung Cell. Mol. Physiol..

[57] Wolff V., Schlagowski A.I., Rouyer O., Charles A.L., Singh F., Auger C., Schini-Kerth V., Marescaux C., Raul J.S., Zoll J. (2015). Tetrahydrocannabinol induces brain mitochondrial respiratory chain dysfunction and increases oxidative stress: A potential mechanism involved in cannabis-related stroke. BioMed Res. Int..

[58] Abboussi O., Tazi A., Paizanis E., El Ganouni S. (2014). Behavior, Chronic exposure to WIN55, 212-2 affects more potently spatial learning and memory in adolescents than in adult rats via a negative action on dorsal hippocampal neurogenesis. Pharmacol. Biochem. Behav..

[59] Williams B.P., Park J.K., Alberta J.A., Muhlebach S.G., Hwang G.Y., Roberts T.M., Stiles C.D. (1997). A PDGF-regulated immediate early gene response initiates neuronal differentiation in ventricular zone progenitor cells. Neuron.

[60] Turnley A.M., Faux C.H., Rietze R.L., Coonan J.R., Bartlett P.F. (2002). Suppressor of cytokine signaling 2 regulates neuronal differentiation by inhibiting growth hormone signaling. Nat. Neurosci..

[61] Ohnuma S.-i., Philpott A., Harris W.A. (2001). Cell cycle and cell fate in the nervous system. Curr. Opin. Neurobiol..

[62] Martinsson-Ahlzén H.-S., Liberal V., Grünenfelder B., Chaves S.R., Spruck C.H., Reed S.I. (2008). Cyclin-dependent kinase-associated proteins Cks1 and Cks2 are essential during early embryogenesis and for cell cycle progression in somatic cells. Mol. Cell. Biol..

[63] Ardehali M.B., Damle M., Perea-Resa C., Blower M.D., Kingston R.E. (2021). Elongin A associates with actively transcribed genes and modulates enhancer RNA levels with limited impact on transcription elongation rate in vivo. J. Biol. Chem..

[64] Sales A.J., Guimarães F.S., Joca S.R.L. (2020). CBD modulates DNA methylation in the prefrontal cortex and hippocampus of mice exposed to forced swim. Behav. Brain Res..

[65] Li Y., Nichols M.A., Shay J.W., Xiong Y. (1994). Transcriptional repression of the D-type cyclin-dependent kinase inhibitor p16 by the retinoblastoma susceptibility gene product pRb. Cancer Res..

[66] Dalterio S., Steger R., Mayfield D., Bartke A. (1984). Behavior, Early cannabinoid exposure influences neuroendocrine and reproductive functions in mice: II. Postnatal effects. Pharmacol. Biochem. Behav..

[67] Narisawa S., Hecht N.B., Goldberg E., Boatright K.M., Reed J.C., Millán J.L. (2002). Testis-specific cytochrome c-null mice produce functional sperm but undergo early testicular atrophy. Mol. Cell. Biol..

[68] Hirakawa I., Miyagawa S., Katsu Y., Kagami Y., Tatarazako N., Kobayashi T., Kusano T., Mizutani T., Ogino Y., Takeuchi T. (2012). Gene expression profiles in the testis associated with testis-ova in adult Japanese medaka (*Oryzias latipes*) exposed to 17α-ethinylestradiol. Chemosphere.

[69] Wang H., Wang Q., Zhao X.-F., Liu P., Meng X.-H., Yu T., Ji Y.-L., Zhang H., Zhang C., Zhang Y. (2010). Cypermethrin exposure during puberty disrupts testosterone synthesis via downregulating StAR in mouse testes. Arch. Toxicol..

[70] Cogliati S., Lorenzi I., Rigoni G., Caicci F., Soriano M.E. (2018). Regulation of Mitochondrial Electron Transport Chain Assembly. J. Mol. Biol..

[71] Willett C.S., Burton R.S. (2004). Evolution of interacting proteins in the mitochondrial electron transport system in a marine copepod. Mol. Biol. Evol..

