# Supplementary material for: Transcriptional Alterations Induced by Delta-9 Tetrahydrocannabinol in the Brain and Gonads of Adult Medaka
Source: J Xenobiot. 2023 May 30;13(2):237–51. doi: 10.3390/jox13020018 (PMC10303197; doi:10.3390/jox13020018)
Supplement: Supplementary file 1 [file jox-13-00018-s001.zip › jox-2380770-supplementary.pdf]

Transcriptional Alterations Induced by Delta-9 Tetrahydrocannabinol in the Brain and Gonads of Adult Medaka

Marlee Vassall[1], Sourav Chakraborty[1], Yashi Feng[1], Mehwish Faheem [1], Xuegeng Wang[1,2], Ramji Kumar Bhandari[1,‡]

1) Department of Biology, University of North Carolina Greensboro, NC 27412, United States
2) Institute of Modern Aquaculture Science and Engineering, College of Life Sciences, South China Normal University, Guangzhou 510631, China

*Corresponding Author: rkbhanda@uncg.edu

Supplemental Figures

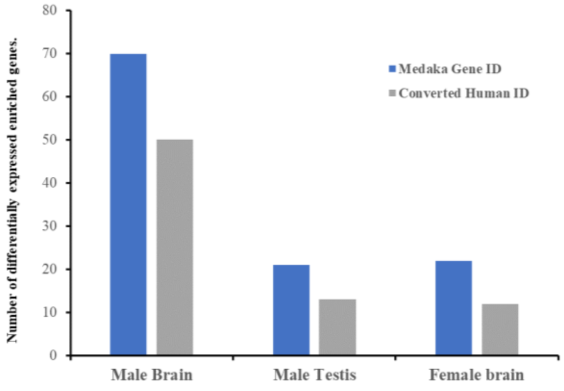

Supplementary Figure S1. The human ortholog of medaka genes in the male brain, testis, and female brain. Y axis indicates the number of differentially expressed genes enriched.

2A

Target name	Term ID	P(adj)	$-log_{10}$ (Padj)
structural constituent of muscle	GO:0008307	3.234×10^{-7}	
oxidoreduction-driven active transmembrane transpor...	GO:0015453	3.076×10^{-4}	
primary active transmembrane transporter activity	GO:0015399	1.803×10^{-3}	
cytochrome-c oxidase activity	GO:0004129	4.341×10^{-3}	
electron transfer activity	GO:0009055	5.034×10^{-3}	
oxidoreductase activity, acting on a heme group of do...	GO:0016675	5.146×10^{-3}	
proton transmembrane transporter activity	GO:0015078	5.649×10^{-3}	
structural molecule activity	GO:0005198	6.276×10^{-3}	
active ion transmembrane transporter activity	GO:0022853	1.520×10^{-2}	
active transmembrane transporter activity	GO:0022804	1.892×10^{-2}	
creatine kinase activity	GO:0004111	3.565×10^{-2}	
phosphotransferase activity, nitrogenous group as acc...	GO:0016775	4.984×10^{-2}	

2B

Target name	Term ID	P(adj)	$- log_{10}$ (Padj)
muscle contraction	GO:0006936	2.332×10^{-7}	
ATP metabolic process	GO:0046034	5.975×10^{-7}	
muscle system process	GO:0003012	2.284×10^{-6}	
generation of precursor metabolites and energy	GO:0006091	1.307×10^{-5}	
actin-myosin filament sliding	GO:0033275	1.631×10^{-4}	
oxidative phosphorylation	GO:0006119	1.102×10^{-3}	
proton transmembrane transport	GO:1902600	1.842×10^{-3}	
aerobic electron transport chain	GO:0019646	2.231×10^{-3}	
mitochondrial ATP synthesis coupled electron transport	GO:0042775	3.453×10^{-3}	
ATP synthesis coupled electron transport	GO:0042773	3.453×10^{-3}	
striated muscle contraction	GO:0006941	3.613×10^{-3}	
nucleoside diphosphate phosphorylation	GO:0006165	4.675×10^{-3}	

2C

Target name	Term ID	P(adj)	$-log_{10}$ (Padj)
contractile fiber	GO:0043292	6.239×10^{-7}	
myofilament	GO:0036379	6.383×10^{-7}	
sarcomere	GO:0030017	4.462×10^{-6}	
myofibril	GO:0030016	9.320×10^{-6}	
striated muscle thin filament	GO:0005865	3.515×10^{-5}	
respiratory chain complex	GO:0098803	4.514×10^{-4}	
cytochrome complex	GO:0070069	4.718×10^{-4}	
respirasome	GO:0070469	7.479×10^{-4}	
cytoplasm	GO:0005737	7.672×10^{-4}	
supramolecular fiber	GO:0099512	1.870×10^{-3}	
supramolecular polymer	GO:0099081	2.018×10^{-3}	
mitochondrial inner membrane	GO:0005743	2.974×10^{-3}	

Supplementary Figure S2. The top 12 enrichments of the biological processes from the Gene Ontology enrichment annotation of male brain from THC-exposed medaka. 2A) Enrichment of biological processes. 2B) Enrichment of molecular function. 2C) Enrichment of cellular components.

3A

Target name	Term ID	P(adj)	$-\log_{10}$ (Padj)
oxidoreduction-driven active transmembrane transpor...	GO:0015453	1.362×10^{-5}	
cytochrome-c oxidase activity	GO:0004129	2.604×10^{-5}	
oxidoreductase activity, acting on a heme group of do...	GO:0016675	3.091×10^{-5}	
active ion transmembrane transporter activity	GO:0022853	5.218×10^{-5}	
electron transfer activity	GO:0009055	1.335×10^{-4}	
proton transmembrane transporter activity	GO:0015078	1.468×10^{-4}	
active transmembrane transporter activity	GO:0022804	4.370×10^{-4}	
primary active transmembrane transporter activity	GO:0015399	5.725×10^{-4}	
inorganic cation transmembrane transporter activity	GO:0022890	3.091×10^{-3}	
cation transmembrane transporter activity	GO:0008324	4.646×10^{-3}	
inorganic molecular entity transmembrane transporter...	GO:0015318	8.086×10^{-3}	
ion transmembrane transporter activity	GO:0015075	1.675×10^{-2}	

3B

Target name	Term ID	P(adj)	$-\log_{10}$ (Padj)
aerobic electron transport chain	GO:0019646	1.161×10^{-4}	
mitochondrial electron transport, cytochrome c to oxy...	GO:0006123	1.589×10^{-4}	
ATP synthesis coupled electron transport	GO:0042773	1.656×10^{-4}	
mitochondrial ATP synthesis coupled electron transport	GO:0042775	1.656×10^{-4}	
ATP metabolic process	GO:0046034	2.141×10^{-4}	
respiratory electron transport chain	GO:0022904	3.578×10^{-4}	
oxidative phosphorylation	GO:0006119	8.341×10^{-4}	
electron transport chain	GO:0022900	1.967×10^{-3}	
aerobic respiration	GO:0009060	2.782×10^{-3}	
generation of precursor metabolites and energy	GO:0006091	3.983×10^{-3}	
cellular respiration	GO:0045333	6.312×10^{-3}	
energy derivation by oxidation of organic compounds	GO:0015980	2.232×10^{-2}	

3C

Target name	Term ID	P(adj)	$-\log_{10}$ (Padj)
cytochrome complex	GO:0070069	5.802×10^{-7}	
respiratory chain complex	GO:0098803	1.647×10^{-5}	
respirasome	GO:0070469	2.487×10^{-5}	
respiratory chain complex IV	GO:0045277	4.049×10^{-5}	
mitochondrial respiratory chain complex III	GO:0005750	2.740×10^{-3}	
respiratory chain complex III	GO:0045275	2.740×10^{-3}	
mitochondrial inner membrane	GO:0005743	1.286×10^{-2}	
organelle inner membrane	GO:0019866	2.018×10^{-2}	
envelope	GO:0031975	3.762×10^{-2}	
organelle envelope	GO:0031967	3.762×10^{-2}	

Supplementary Figure S3. Top 12 enrichments of the biological processes from the Gene Ontology enrichment annotation of testis from Δ⁹-THC -exposed medaka. 3A) Enrichment of biological processes. 3B) Enrichment of molecular function. 3C) Enrichment of cellular component.

4A

Target name	Term ID	P(adj)	$-\log_{10}$ (Padj)
cyclin-dependent protein serine/threonine kinase regu...	GO:0016538	2.588×10^{-4}	
protein kinase regulator activity	GO:0019887	2.091×10^{-2}	
kinase regulator activity	GO:0019207	3.043×10^{-2}	

4B

regulation of cyclin-dependent protein serine/threoni...	GO:0000079	1.228×10^{-2}	
regulation of cyclin-dependent protein kinase activity	GO:1904029	1.394×10^{-2}	
mitotic cell cycle phase transition	GO:0044772	4.180×10^{-2}	

4C

cyclin-dependent protein kinase holoenzyme complex	GO:0000307	1.202×10^{-4}	
cyclin A2-CDK2 complex	GO:0097124	2.369×10^{-4}	
serine/threonine protein kinase complex	GO:1902554	1.092×10^{-3}	
protein kinase complex	GO:1902911	1.794×10^{-3}	
transferase complex, transferring phosphorus-containi...	GO:0061695	2.902×10^{-2}	

Supplementary Figure S4. Gene Ontology enrichment annotation of the female brain. 4A) Enrichment of biological processes. 4 B) Enrichment of molecular function. 4C) Enrichment of cellular components.

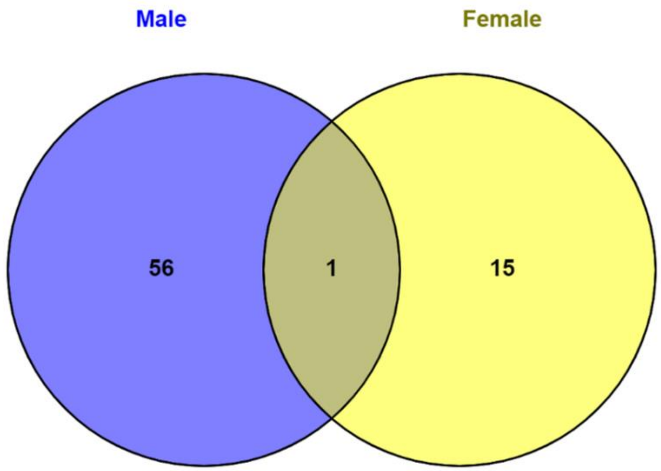

Supplementary Figure S5. Venn diagram of DEG in males and female showed a unique set of genes differentially expressed in THC-exposed medaka
